# Successful surgical closure and continence rate of obstetric fistula in Africa: systematic review and meta-analysis

**DOI:** 10.3389/fgwh.2023.1188809

**Published:** 2023-10-03

**Authors:** Henok Kumsa, Esuyawkal Mislu, Mulugeta Wedaje Arage, Atitegeb Abera, Tilahun Hailu, Lebeza Alemu Tenaw

**Affiliations:** ^1^ School of Midwifery, College of Midwifery, Woldia University, Woldia, Ethiopia; ^2^ School of Public Health, College of Midwifery, Woldia University, Woldia, Ethiopia

**Keywords:** obstetric fistula, successful surgical closure, continent rate, combined VVF and RVF, Africa

## Abstract

**Background:**

A female genital fistula is an abnormal connection between a woman's reproductive tract and her urinary tract or rectum. While numerous studies have aimed to determine the success rate of obstetric fistula closure in different health settings, there remains a significant scarcity of data on closure success rates and incontinence rates for various types of fistulas at the regional and sub-regional levels. The success rate reflects the continent's healthcare setup in regard to the World Health Organization standards. Thus, this study aims to determine the success of surgical closure and the continence rate of obstetric fistula in Africa.

**Methods:**

This systematic review and meta-analysis review includes studies conducted up to February 2023. Search engines like EMBBASE, Medline, Google, PubMed, Google Scholar, African Journals Online, and ScienceDirect databases were utilized to find articles. The Joanna Briggs Institute critical evaluation checklist was used to evaluate the quality of our review, which was conducted in accordance with PRISMA criteria. Heterogeneity was indicated by a *p*-value for I^2^ statistics of less than 0.05. Publication bias was assessed using the Egger regression asymmetry test. Data were entered into Microsoft Excel and analyzed using STATA 16.

**Result:**

This review includes 85 studies. A total of 24 countries from East, West, Central, North, and Southern African sub-regions were included. The overall pooled estimated rate of successful obstetric fistula closure is 86.15 (95% CI: 83.88–88.42). Moreover, the pooled estimated rate of successfully closed vesico-vaginal fistulas but with ongoing or residual incontinence (wet) was revealed as 13.41% (95% CI: 11.15–15.68). The pooled estimated rate of successfully closed rectovaginal fistulas and combined VVF and RVF are 91.06% (95% CI: 86.08–96.03) and 62.21% (95% CI: 48.94–75.49), respectively.

**Conclusions:**

The rate of successful obstetric fistula closure in Africa is 86.15, which is higher than the WHO target. However, the surgical closure rate of a combined VVF and RVF is 62.2%, which is significantly lower than the WHO target.

## Introduction

A female genital fistula is an abnormal connection between a woman's reproductive tract and her urinary tract or rectum (1). The World Health Organization (WHO) describes vaginal fistulas as the single most cause adverse consequence of neglected childbirth (2). Vaginal fistulas are common in poor countries, mostly in South Asian and Sub-Saharan African nations, where the social norm promotes marriage at a young age, frequently soon after the girl's first period between the ages of 9 and 15. The first pregnancy occurs soon after marriage in many of these situations (3, 4).

Although the global burden of the disease is not exactly known, it is estimated to be 3 million, with 30,000 to 130,000 new cases added each year. Additionally, over 2 million women are living with untreated obstetric fistula in developing countries (5, 6). Fistulas can be categorized, depending on the affected anatomical regions, as rectovaginal fistulas (between the genital tract and the rectum) or vesicovaginal fistulas (between the genital tract and the urinary tract) (7, 8).

Female genital fistulas can occur because of obstetric complications, gynecological procedures, and trauma. Even in expert hands, genital tract injuries are known to occur during gynecological procedures (2, 9). The incidence and the etiology of genital fistulas show geographical variation. In developed countries, 83.2% of fistulas occur following surgery, whereas in low-resourced countries, 95.2% are associated with childbirth when women do not have access to timely emergency obstetric care (10).

The consequences of obstetric fistula are far greater than the visible medical condition. Women with obstetric fistulas have a persistent odor associated with continued urine and/or stool leakage, putting them at risk of health problems and ostracization by their husbands and community, with up to 52% of affected women facing divorce. It is also linked to a higher risk of mental health disorders, with nearly 97% of affected women screening positive for potential mental health disorders. Moreover, obstetric fistula affects economically vulnerable women and garners little attention on the global health stage (11, 12).

With the extensive effects of fistulas on women's health, surgical closure of the fistula is critical to the woman's overall well-being; without it, the likelihood of fistula resolution is almost null, except in the rare cases of early fistula closure by immediate catheterization (13, 14). The WHO established the ideal range of repair outcomes as less than 15% for failed fistula closure and less than 10% for incontinence after successful closure (15). However, there are not many facilities that have the tools and staff that are qualified to conduct fistula closure. Moreover, surgical closure of obstetric fistula does not ensure the patient will have a satisfactory outcome and be able to resume her normal activities; up to 42% of fistula repairs fail, and up to 67% of successful surgical closures result in residual incontinence (16, 17).

Interestingly, the degree of success in obstetric fistula treatment varies depending on the patient (fistula type and location) and health system (staff training, surgical expertise) (18, 19). Although numerous studies have attempted to identify the success rate of fistula repair in African nations, which ranges from 42% in Angola to 97% in Malawi (16, 20); there is a lack of data on the success rate of fistula closure at the continental and sub-region level to indicate the health care setting status. Therefore, this study aims to assess the successful surgical closure rate of obstetric fistula (VVF, RVF, and combined VVF and RVF) in Africa.

## Methods

### Study design and setting

This systematic review and meta-analysis includes cross-sectional, cohort, and randomized control trial studies conducted in Africa. A comprehensive review and analysis of data from computerized databases was conducted to determine the success rate of fistula closure in Africa.

### Search strategy

For this review, relevant articles were searched with different search strategies. Published articles were searched from online databases such as EMBBASE, Medline, Google, PubMed, Google Scholar, African Journals Online, and ScienceDirect databases. In addition, we extended our search by retrieving and extracting potential articles from reference lists of eligible articles. A recommended PRISMA guideline was strictly followed throughout this review. Similarly, the quality of our systematic review and meta-analysis was assessed by the Joanna Briggs Institute critical appraisal checklist (21).

Searching was conducted using Medical Subject Heading (MeSH) terms related to successful fistula closure or repair. MeSH terms enabled us to select related research articles. We conducted the search for terms using Boolean operators “AND” and “OR,” both separately and in combinations. The search terms for the rate of successful fistula closure were ((Fistula) OR (Obstetrics Fistula)) OR (Urinary Fistula)) OR (Vesicovaginal fistula)) OR (Ureterovaginal fistula)) OR (Rectovaginal fistula)) AND (Intervention)) OR (surgical Repair)) OR (surgical Closure)) OR (outcomes)) OR (Successful surgical closure outcomes)) AND (Africa). The search was also made by combining the above search terms with the names of all countries included in Africa. All the search terms are included in the Supplementary material.

### Eligibility criteria

#### Inclusion criteria

There were no restrictions on research design. Cross-sectional, cohort, and randomized control trial studies conducted on the success of fistula closure in Africa were included. Only articles reported in the English language and studies conducted before February 2023 were included. The original articles assessed mainly obstetric fistula closure success rates.

#### Exclusion criteria

This review does not consider studies focused on fistulas caused by gynecologic surgery or trauma. We excluded articles without full text as they cannot be assessed for their quality. Agreements on the inclusion and exclusion of the articles were held through the participation of all authors.

### Quality assessment

The quality of the studies were assessed using the Joana Brigg's Institute (JBI) critical appraisal checklist. The checklists were available online and it was designed separately for cross-sectional, and cohort studies. We used more than one checklist depending on the study design of the published articles. Quality assessment was carried out by all authors. The critical appraisal checklist has eight questions overall; articles with a score of 5 or more out of 8 in the JBI criteria were considered to be of good quality and were included in the review. Any critical appraisal discrepancies among reviewers were resolved through discussion with the third-party reviewer. The table containing the quality appraisal scores is available in the Supplementary material.

### Heterogeneity and publication bias

The heterogeneity test of included studies was assessed using the *I*^2^ statistics. Heterogeneity was indicated by a *P*-value for *I*^2^ statistics of less than 0.05. The findings of the *I*^2^ test were classified as having Low (25%), Moderate (50%), and High (75%) heterogeneity (22). Moreover, the Egger regression asymmetry test was used to evaluate publication bias (23, 24). When the *p*-value for the Egger test is less than 0.05, publication bias is shown. Additionally, Duval and Tweedie's nonparametric trim and fill analysis using the random effect analysis was conducted to account for publication bias (25).

### Outcome Variable

The outcome variable for this review was the rate of successful obstetric fistula closure. This variable has two categories, dichotomized as yes/no. We searched articles that measure and assess this outcome variable.

### Data extraction and management

After the data had been screened by title, abstract and reviewing the articles. The data were prepared/extracted in Microsoft excel form, checked and evaluated by all authors. The data extraction format consists of name of first author, title, publication and study year, study country and sub-region, study design, sample size and rate of successful surgical closure of obstetrics fistula types (The extracted data is available in Supplementary file).

### Registration and protocol

This review has not previously registered or prepared a protocol. As a result, no adjustments have been made.

### Data processing and analysis

The extracted data were exported into Stata version 16 for analysis (meta-analysis). The heterogeneity of included studies was assessed by *I*^2^-statics. Publication bias across studies were assessed subjectively by observing the funnel plot and objectively by considering Egger's test estimates at a 5% level of significance. Forest plots were used to estimate the pooled prevalence and effect size of each study. The estimates were presented with a 95% confidence interval. The size of each box indicated the weight of the study, while each crossed line refers to the 95% confidence interval. Subgroup analysis was done by sub-region, which enables the assessment of how successful fistula closure varies across the region of Africa.

## Result

### Study selection

**Figure 1:**
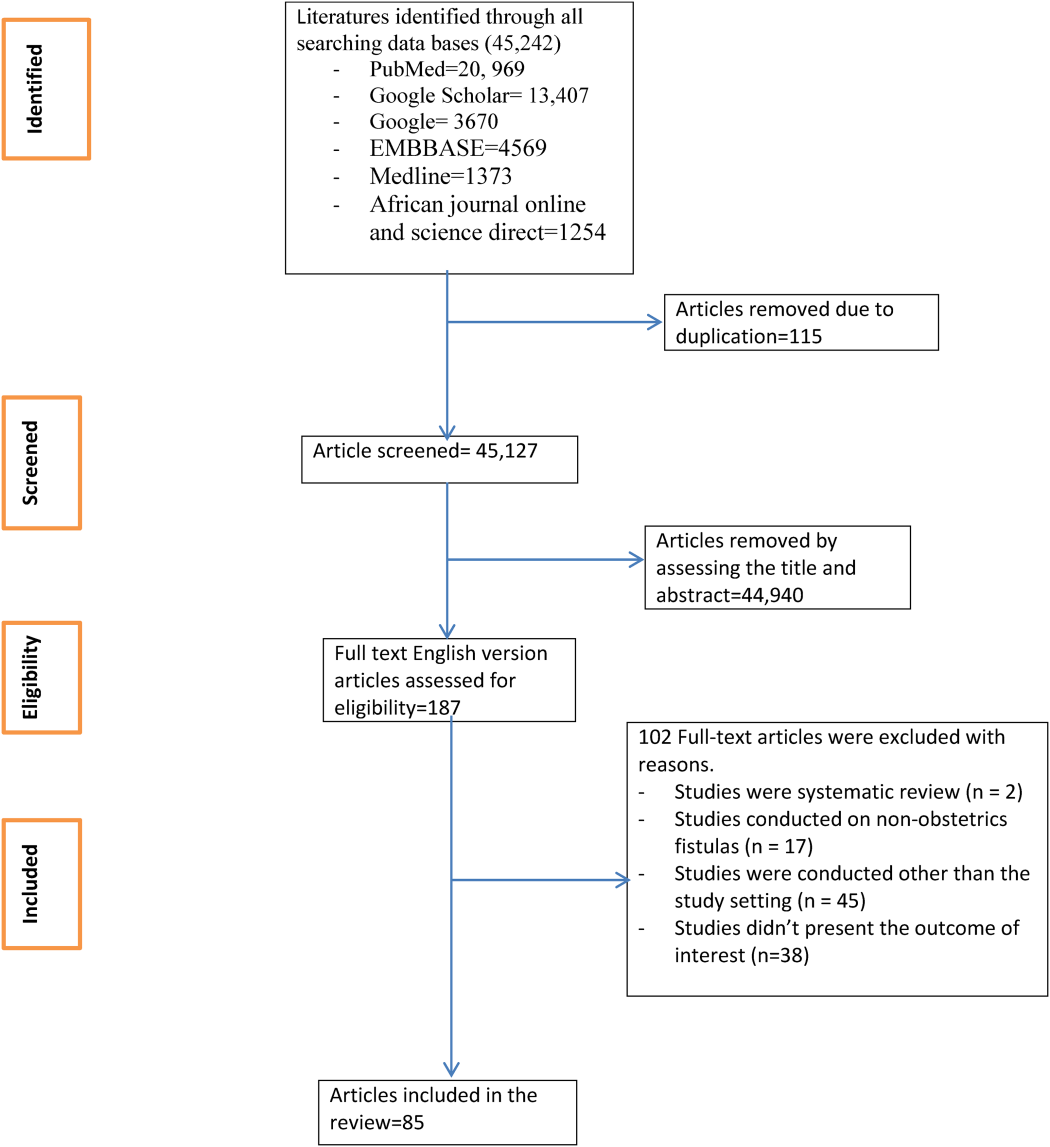
Flow chart of study selection for systematic review and meta-analysis of successful surgical closure of obstetric fistula in Africa.

This systematic review and meta-analysis includes published studies on the surgical closure of obstetric fistula in Africa. A total of 45,242 records were retrieved through electronic database searching, and 85 articles were included to estimate the pooled rate of successful surgical closure of different types of obstetric fistula (Figure 1).

### Characteristics of included studies

**Figure 2:**
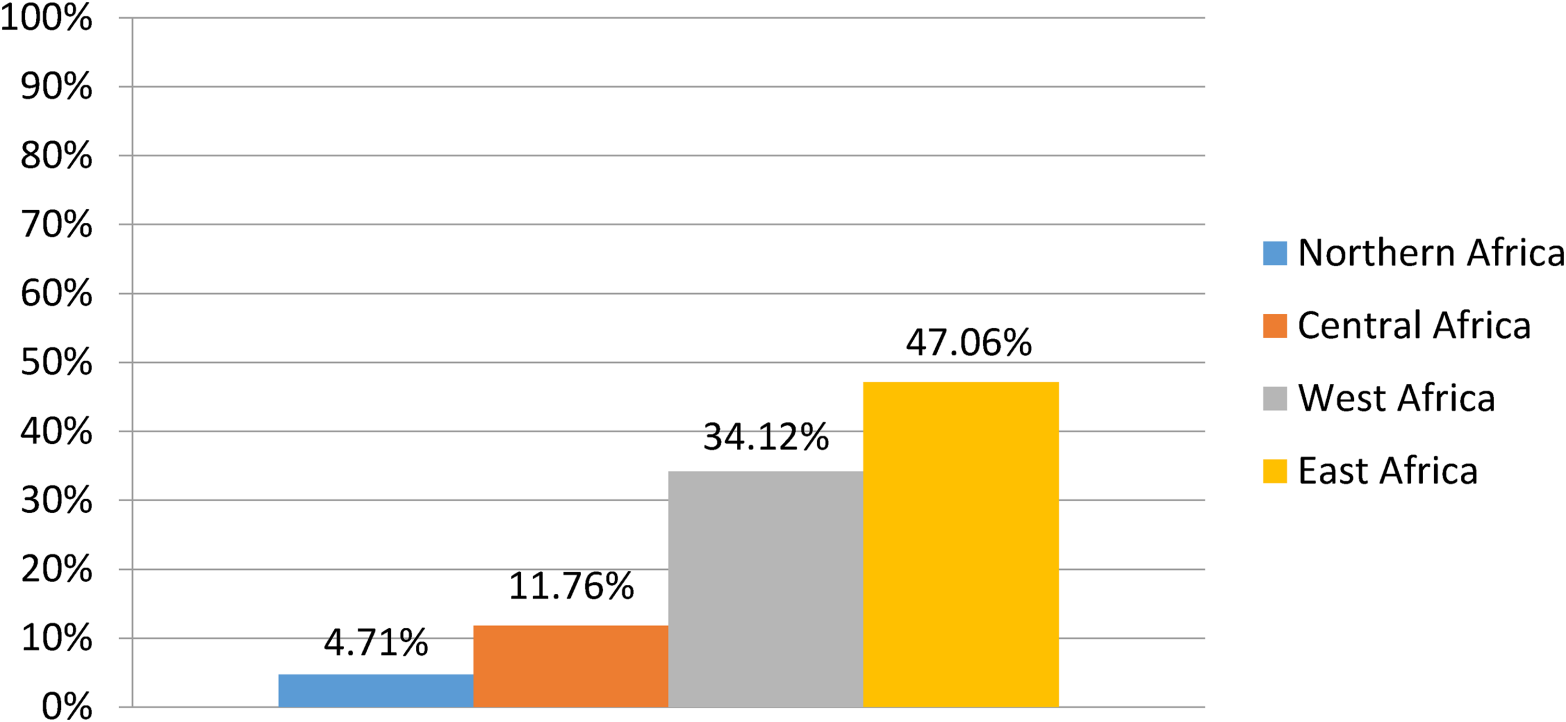
Regional representation of the included studies.

This review includes studies from 24 different African nations. Of the total, 4 (4.71%) were from Northern Africa (26–29), 10 (11.76%) were from Central Africa (13, 16, 30–37), 31 (34.12%) were from West Africa (38–68), and 40 (47.06%) (19, 69–107) were from East Africa (Figure 2). The majority of the included articles were from Sub-Saharan African countries such as Ethiopia, Nigeria, and the Democratic Republic of Congo. Except for a single cross-sectional study from Ethiopia (93), all the included articles were longitudinal and randomized control trial studies.

## Rate of successful surgical closure of obstetric fistula in Africa

**Figure 3:**
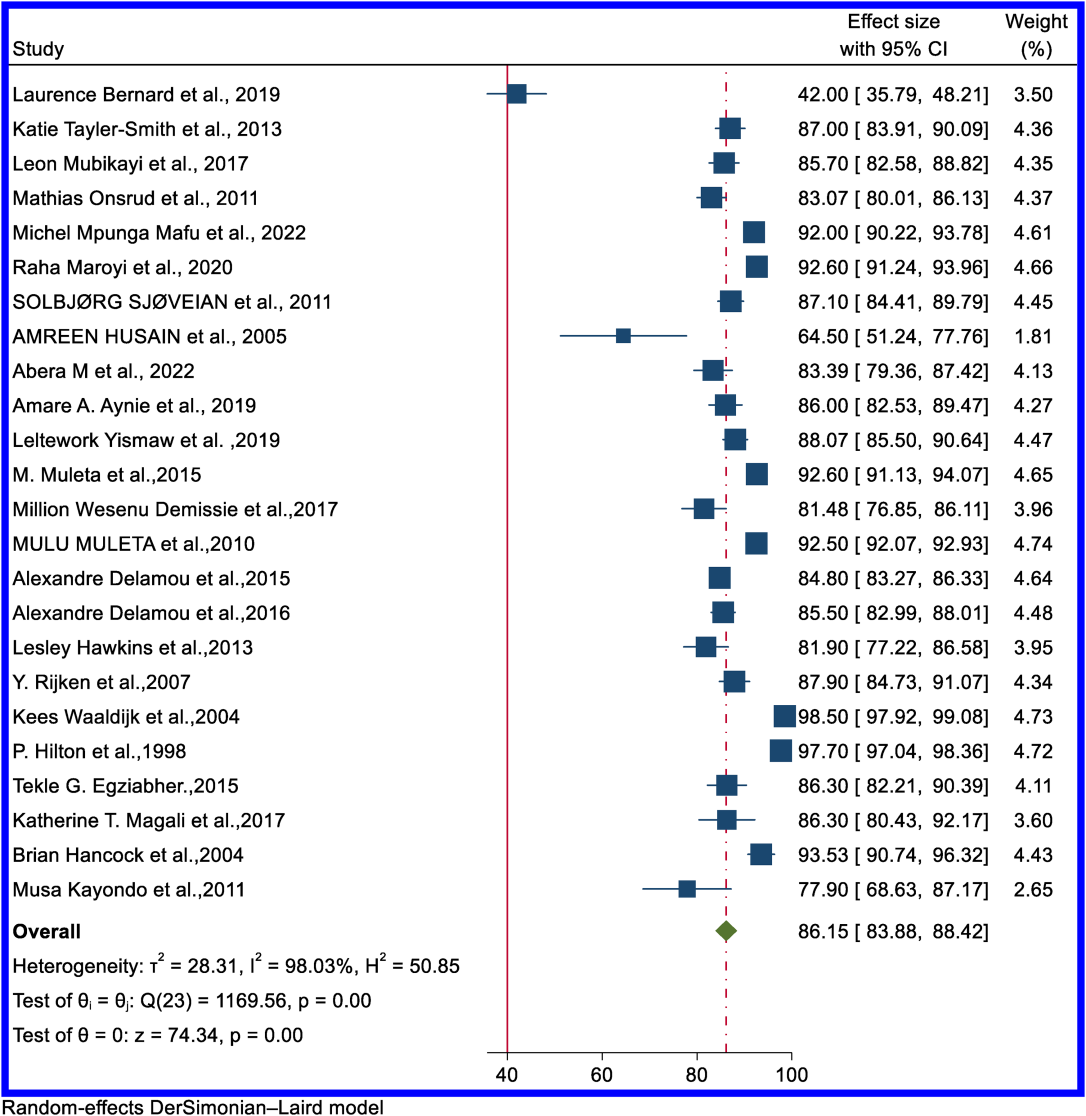
Forest plot of the pooled rate of successful surgical closure of overall obstetrics fistula in Africa.

In some articles, for all types of fistulas (VVF, RVF, UVF, VUF, and combined VVF and RVF), successful closure was reported as successful obstetric fistula closure rate. Therefore, the pooled result of successful surgical closure of obstetric fistula uses “obstetric fistula” as a reference for all the different types of fistulas reported. The rate of successful fistula closure varies by country, from 42% in Angola (16) to 98.5 in Nigeria (52). The pooled estimated rate of the overall successful obstetric fistula surgical closure rate in Africa as reported by 23 articles is 86.15% (95% CI: 83.88–88.42) (Figure 3). The studies included in the meta-analysis demonstrated a substantial degree of heterogeneity (*I*^2 ^= 98.03%; *p*-value = 0.00001). Egger's regression asymmetry test also revealed significant publication bias, with a *p*-value of 0.00001. The figure for funnel plot and trim and fill analysis is available in Supplementary Figures S1 and S2, respectively. After adjustment, the overall pooled successful closure rate after the trim and fill analysis was 81.3% (95% CI: 76.34–86.27).

In sub-regional analysis, West Africa had the highest successful surgical closure rate, at 91.74%; 95% CI: 86.61–96.88), and Central Africa had the lowest, at 84.04% (95% CI: 73.70–88.38). Supplementary Figure S3 contains a forest plot for the sub-regional distribution of the overall successful obstetric fistula surgical closure rate.

### Rate of successful surgical closure of VVF in Africa

**Figure 4:**
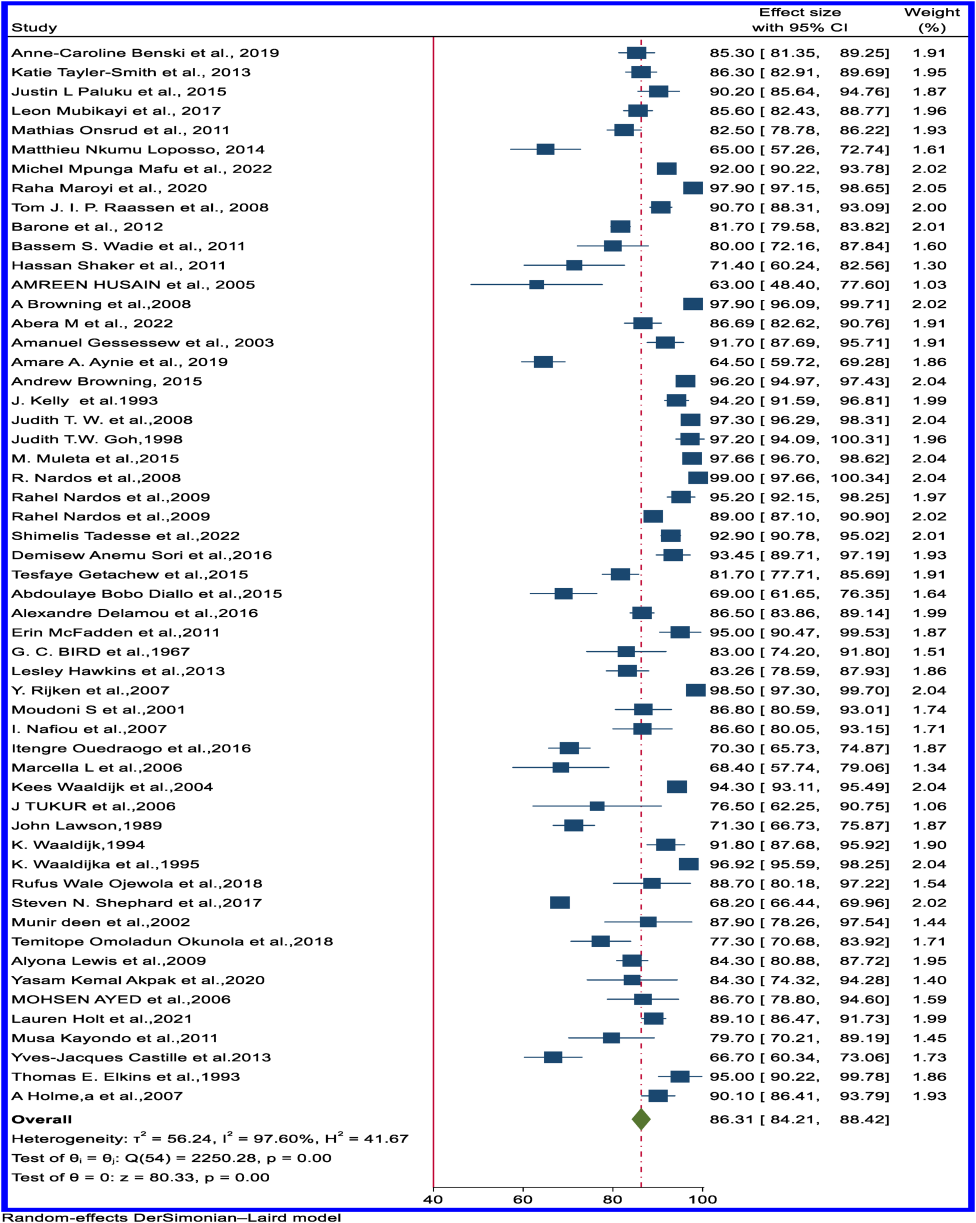
Forest plot of the pooled rate of successful surgical closure of obstetric VVF with unknown status of incontinence in Africa.

The successful surgical closure rate of VVF with unknown urinary incontinence status ranged from 63% in Eretria (72) to 100% in Liberia (46) and Nigeria (50). The pooled estimated rate of successful closure of VVF with unknown incontinence status from 55 articles is 86.31% (95% CI: 84.21–88.42) (Figure 4). The funnel plot is available in Supplementary Figure S4.

In sub-regional analysis, the successful surgical closure rate of VVF with unknown incontinence status was highest, at 90.05% (95%CI: 87.84–92.25), in East Africa and lowest, at 81.60% (95%CI: 75.89–87.31), in West Africa (Table 1). The forest plot of the pooled successful closure rate for VVF with unknown incontinence status based on the sub-regional distribution is available in Supplementary Figure S5.

**Table 1:** Sub-group analysis of the successful surgical closure of obstetric fistula in Africa, 1953-2023.

Subgroup	Number of studies	Total sample	Successful closure rate	95% CI	Heterogeneity	
					*I* ^2^	*p*-value
All types of obstetric fistula reported as single rate (VVF, RVF, UVF, or combined VVF and RVF)						
East Africa	14	18,994	86.75^a^	84.49–89.02	90.36	0.000
West Africa	4	6,540	91.74^a^	86.61–96.88	99.15	0.000
Central Africa	6	4,659	81.04^a^	73.70–88.38	98.2	0.000
Total	23	30,193	86.15^a^	83.88–88.42	98.03	0.000
VVF successfully closed and unknown urinary incontinence status						
East Africa	27	10,897	90.05^b^	87.84–92.25	96.13	0.000
West Africa	18	8,003	81.60^b^	75.89–87.31	98.28	0.000
Northern Africa	4	348	82.25^b^	76.12–83.38	57.01	0.07
Central Africa	6	3,487	86.27^b^	80.07–92.48	97.54	0.000
Total	55	22,735	86.31^b^	84.21–88.42	97.6	0.000
VVF successfully closed with continence (dry) status						
East Africa	23	8,648	75.58^c^	71.50–79.67	95.14	0.000
West Africa	21	10,964	74.51^c^	68.09–80.93	98.7	0.000
Northern Africa	1	114	76.30^c^	68.49–84.11	….	…
Central Africa	8	4,103	82.10^c^	76.42–87.79	96.93	0.000
Total	53	23,829	76.42^c^	73.11–79.73	98.2	0.000
VVF successfully closed but incontinent						
East Africa	22	22,545	18.48^d^	15.22–21.75	96.57	0.000
West Africa	14	7,661	9.33^d^	7.22–11.43	90.47	0.000
Northern Africa	2	214	7.43^d^	2.08–12.79	57.05	0.13
Central Africa	7	4,386	7.52^d^	4.21–10.84	95.5	0.000
Total	47	34,806	13.41^d^	11.15–15.68	97.69	0.000
RVF successfully closed						
Africa	6	612	91.06^e^	86.08–96.03	79.31	0.000
Combined VVF and RVF successfully closed						
Africa	4	250	62.21^f^	48.94–75.49	78.94	0.000

^a^ Rate of successfully closed for all types of fistulas.

^b^ Rate of successfully closed but unknown incontinence status.

^c^ Rate of successfully closed and continent.

^d^ Rate of successfully closed but with residual stress or incontinence.

^e^ Rate of RVF successfully closed.

^f^ Rate of successfully closed combined VVF and RVF.

### Rate of successful surgical closure of VVF based on incontinence/continence outcome

**Figure 5:**
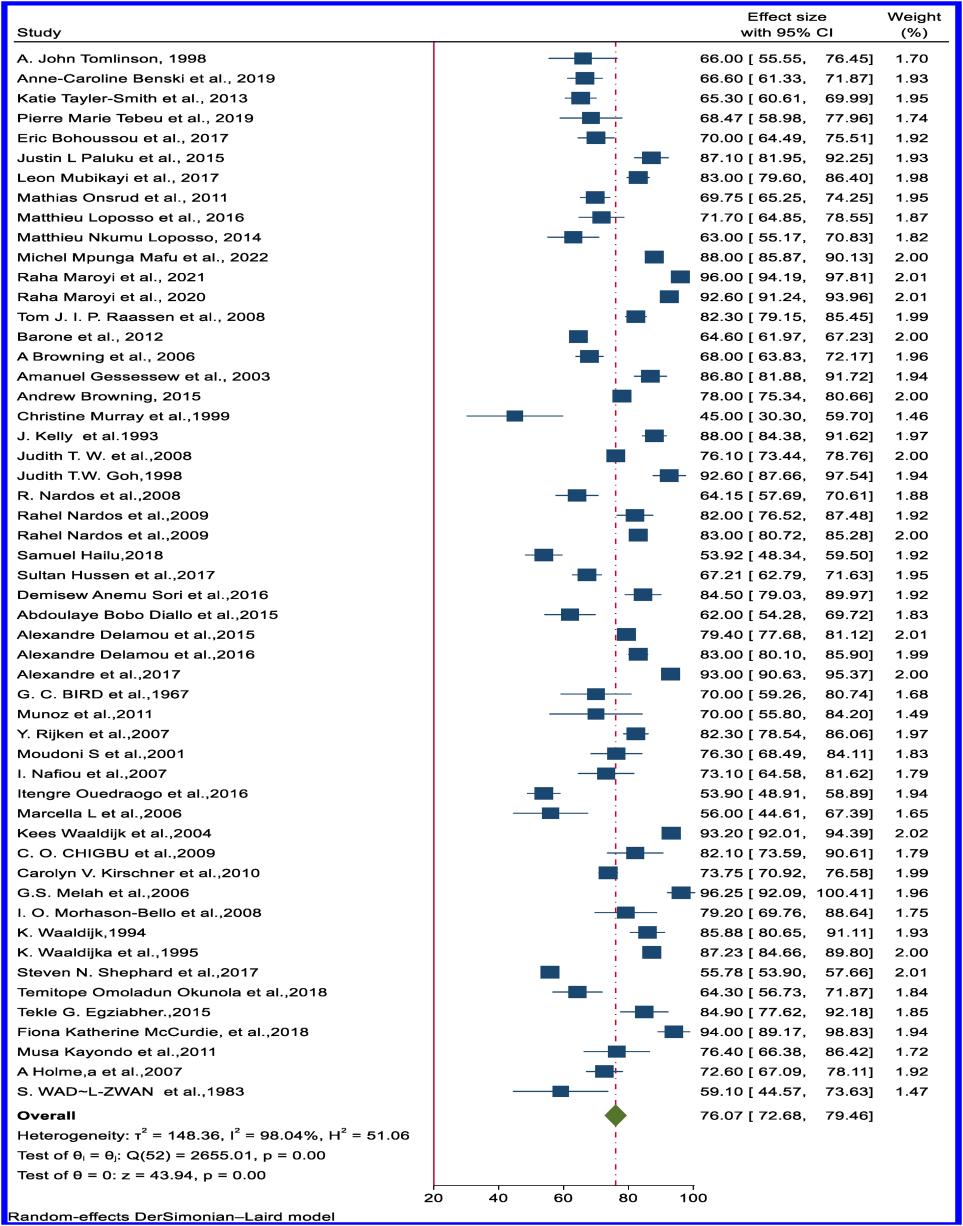
Forest plot of the pooled rate of successfully closed and continent (dry) obstetrics VVF in Africa.

Successful surgeries resulting in continence (dry) ranged from 45% in Ethiopia (79) to 100% in Nigeria (50). The pooled estimated rate of VVF successfully closed and dry from 53 articles was 76.07% (95% CI: 72.68–79.46) (Figure 5). Supplementary Figures S6 and S7 contain the funnel plot figures for VVF successfully closed and dry and VVF successfully closed but with stress or residual incontinence (wet). Sub-regional analysis showed relatively comparable results across the regions (Table 1).

**Figure 6:**
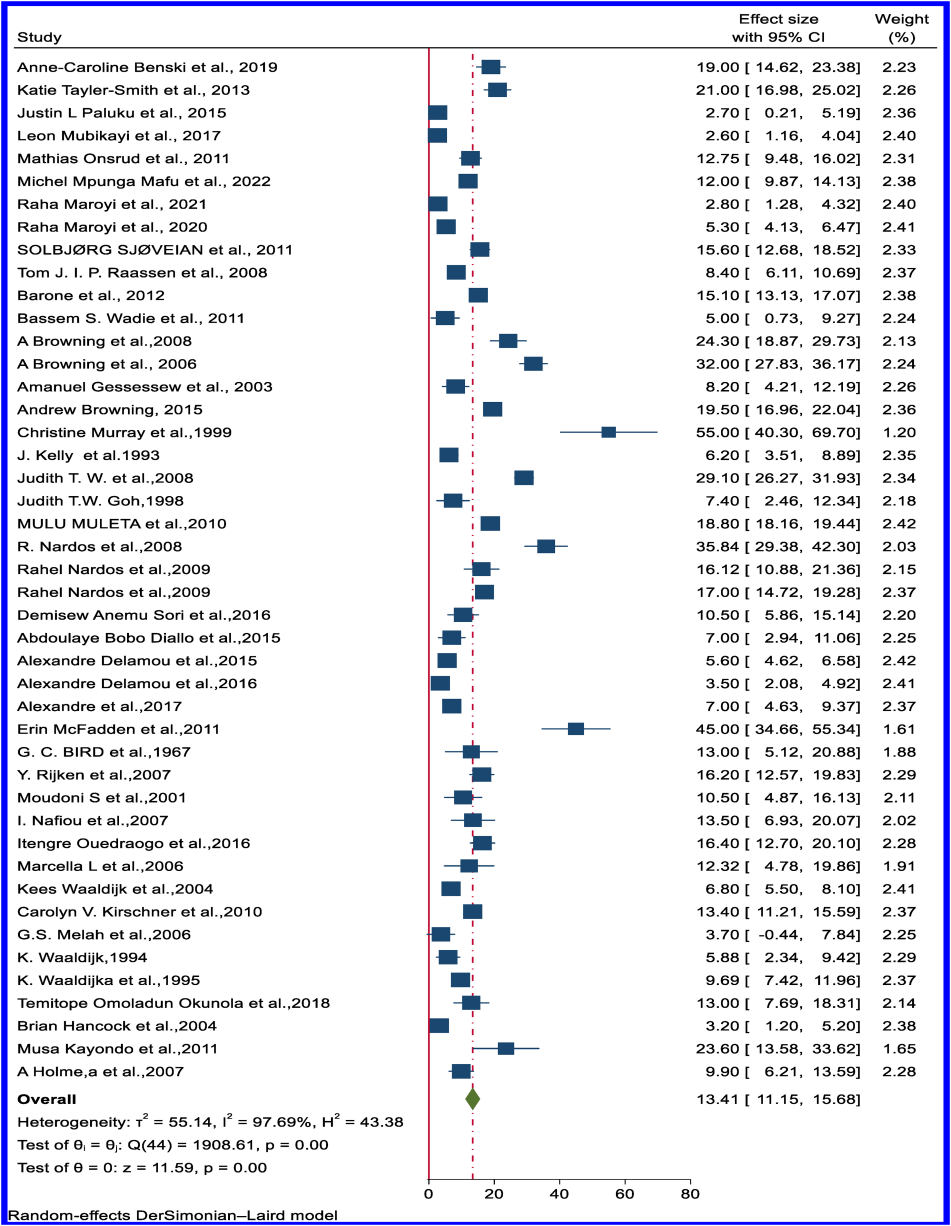
Forest plot of the pooled rate of successfully closed but incontinent (wet) of obstetric VVF in Africa.

Moreover, the pooled estimated rate of VVFs that are closed but have stress or residual incontinence (wet) from 45 articles revealed 13.41% (95% CI: 11.15–15.68) (Figure 6). Sub-regional analysis results show a large variation across the regions: the highest residual stress or incontinence despite successful closure was observed in East Africa, at 18.48% (95% CI: 15.22–21.75), and the lowest was observed in Northern Africa, at 7.43% (95% CI: 2.08−12.79) (Table 1). Supplementary Figures S8 and S9 show the forest plot of the pooled rate of VVF successfully closed and dry and VVF successfully closed but wet based on the sub-regional distribution.

### Rate of successful surgical closure of rectovaginal fistula and combined RVF and VVF

**Figure 7:**
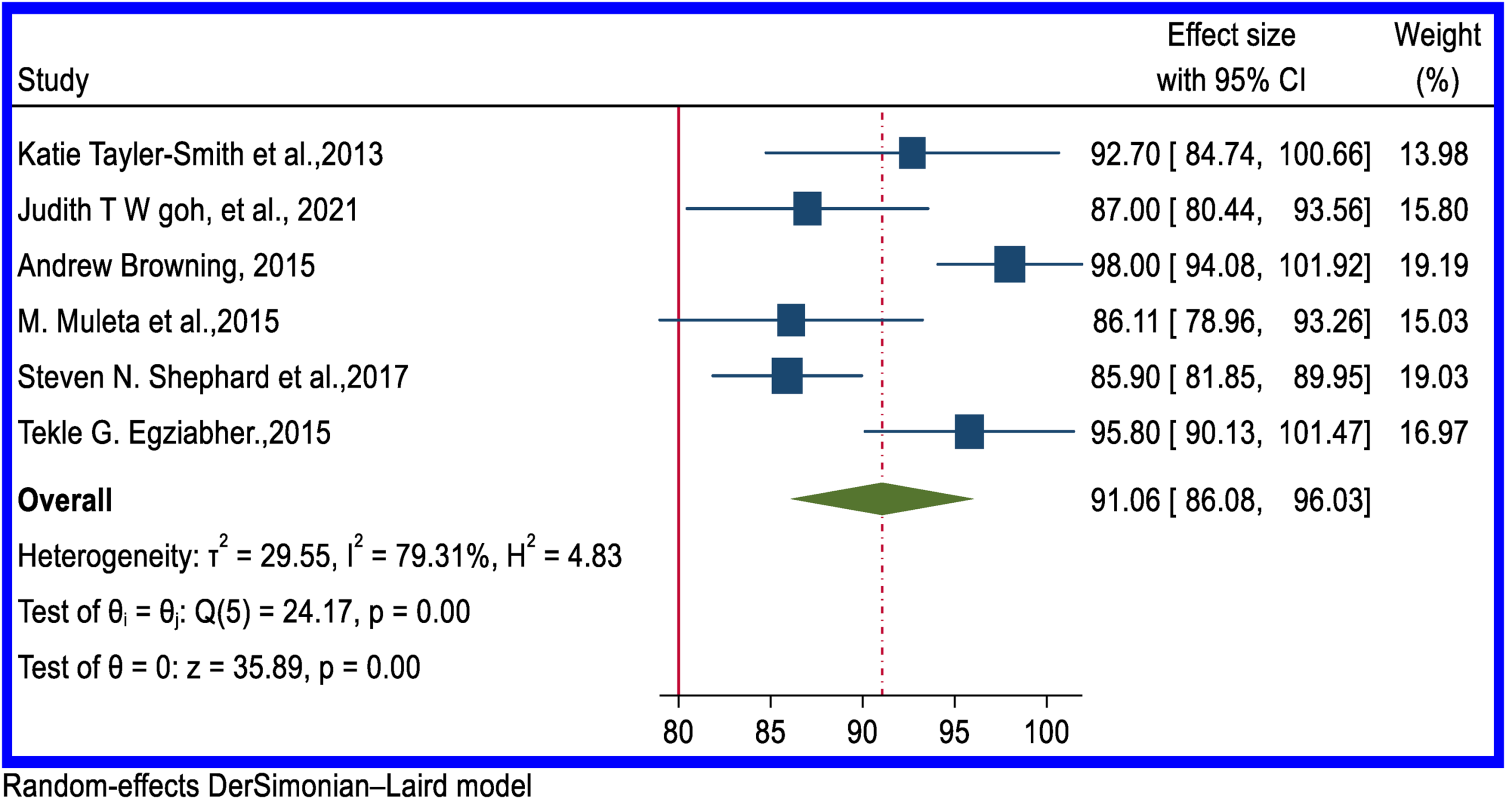
Forest plot of the pooled rate of successful surgical closure of obstetric RVF in Africa.

**Figure 8:**
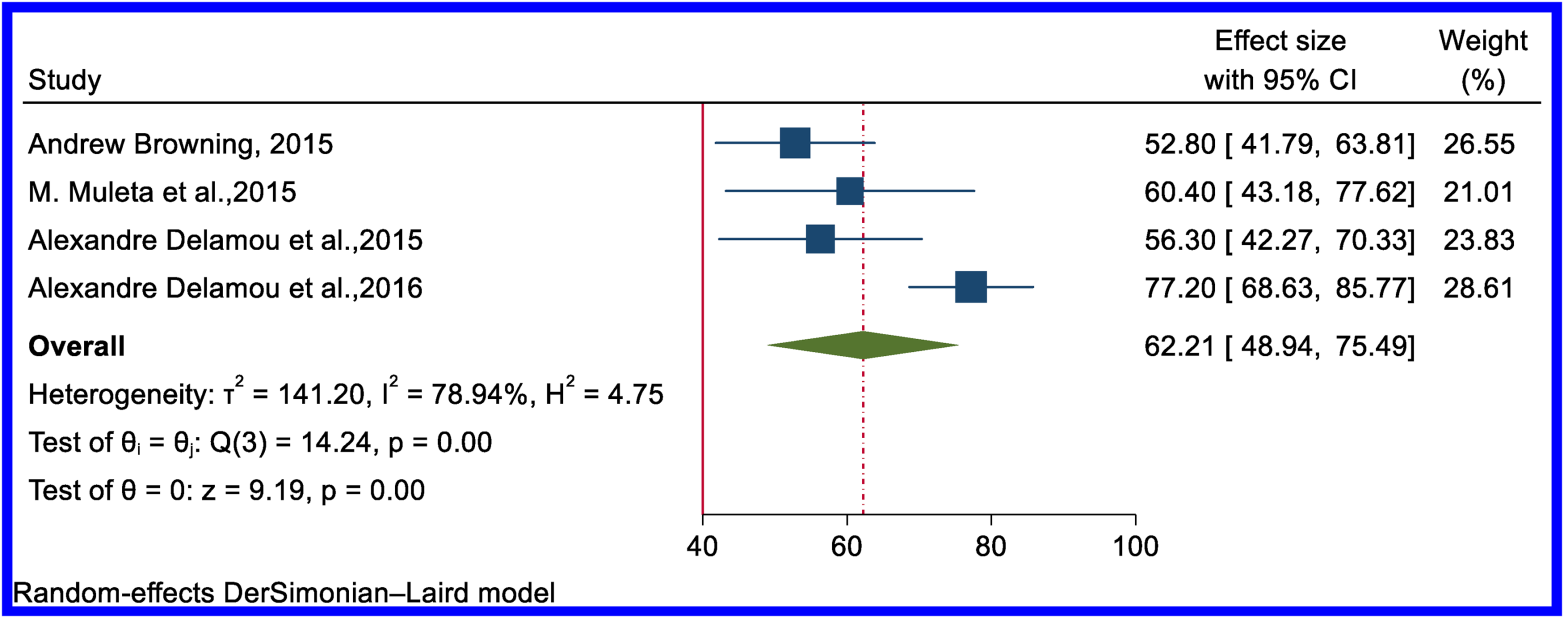
Forest plot of the pooled rate of successful surgical closure of obstetric combined RVF and VVF in Africa.

In all reviewed articles, the success rate for surgical closure of RVF was above 80%, and in five studies, it was 100%. Six studies provided a pooled estimated rate of the successful closure of RVF of 91.06% (95% CI: 86.08–96.03) (Figure 7). From five articles, the overall successful closure rate of combined VVF and RVF was 62.21% (95% CI: 48.94–75.49) (Figure 8). Supplementary Figures S10 and S11 contain the funnel plot figures for the successful closure of RVF and combined VVF and RVF, respectively.

## Discussion

Obstetric fistula is still a public health concern in Africa. However, the public and medical community remain largely unaware of this problem (6). Obstetric fistula closure rate reports across healthcare settings of African countries are varied. Therefore, this systematic review and meta-analysis were conducted to estimate the pooled successful surgical closure rate for different types of obstetric fistulas in Africa.

Our results show that the pooled estimated rate of successful closure of VVF with unknown incontinence status is 86.31% (95% CI: 84.21–88.42). The pooled estimated rate of successfully closed VVF and continent (dry) is 76.07% (95% CI: 72.68–79.46). The pooled estimated rate of successfully closed VVF but with ongoing or residual incontinence (wet) is 13.41% (95% CI: 11.15–15.68). Despite the high rate of successful closure, a significant number of women are faced with ongoing or residual incontinence. This might be because a large number of women live with a fistula for several years before seeking medical assistance, and this might affect the successful surgical closure of obstetric fistulas in the region (69).

In line with the Nepal finding (88%), this review reported a successful surgical closure rate of VVF of 86.31% (95% CI: 84.21–88.42). The sub-regional result also showed a lower successful closure rate than Nepal's finding, except in the East African region, which had a higher success rate (90.05%). A systematic review conducted in both developed and underdeveloped countries showed the rate of successful surgical reconstruction in developed countries was >90% (10, 108). The high success rate was due to the different types of fistulas, as in these countries, small iatrogenic fistulas are more common, which are much easier to cure. Yet, a relatively comparable result was found in underdeveloped countries (10).

This review reveals that the Northern Africa sub-region has a lower success rate (82.25%) than the WHO recommendation, whereas, the East, West, and Central African sub-regions have success rates comparable to or higher than the recommendation. This might be because the studies conducted in Northern Africa were from 1983 to 2011 and had small sample sizes.

The transvaginal route of surgical closure is preferred as it has low morbidity, higher success rates, and minimal complications. Nevertheless, transabdominal VVF closure in correctly chosen individuals yields satisfactory treatment outcomes (109, 110). Moreover, to increase the success rate of closure using a combined abdominovaginal approach with the use of a generous rotational bladder flap for closure of a complex vesicovaginal fistula is vital. Additionally, due to the excellent exposure and healthy, well-vascularized tissue, giant vesicovaginal fistulas have a high success rate on the first attempt (111). Thus, fistula surgeons must consider the best method of closure for complicated fistulas as the first attempt is vital for the success of surgical fistula closure. Moreover, for women who initially present with incontinence, physiotherapy, pelvic floor training, and abdominal wall control are crucial steps to take before surgery (66). Although there isn't enough evidence to support it, encouraged to continue exercise improvement were noted in residual stress incontinence (112).

According to our review, combined VVF and RVF have a lower surgical closure rate than other types of fistulas. Because a combined fistula is indicative of more severe and extensive injuries and more scarring, patients are more likely to have a circumferential VVF, which has worse outcomes. A trial investigation also demonstrated that the use of fibrin glue as an interposition layer during the complex VVF vaginal anatomical closure appears as an alternative to the use of Martius flap interposition (113). Fibrin glue is a valuable resource that can improve the outcome of the closure of VVFs and decrease the time and complexity of the procedure (114). Lastly, difficult and complicated fistulas, experienced surgeons, the establishment of separate fistula surgery units, and appropriate care and expertise are important factors in achieving the desired results (115).

As a limitation, the literature included in this review has a lack of consistency in assessment methods used to investigate obstetric fistula closure and continence rate. In addition, differences in the study population and setting among the included studies might influence the results of this review. Furthermore, the scope of this review was restricted to English-language literature on obstetric fistula. Future review studies that explain factors affecting the successful surgical closure of obstetric fistulas are vital.

## Conclusions and recommendations

Though there is a high rate of successful obstetric fistula closure in Africa, significant numbers of women face residual or ongoing incontinence. Furthermore, the successful surgical closure rate of combined VVF and RVF was found to be considerably below the WHO recommendation.

In Africa, a comprehensive package of fistula care using a dedicated fistula facility or a mobile surgical outreach program might address the problem. Additionally, increased access to timely, quality fistula treatment and comprehensive post-operative care for women with fistulas in Africa is vital to achieving high success rates and lower residual incontinence. Furthermore, wide-scale network-based treatment of fistulas has improved awareness, reduced stigma, increased access to surgery, strengthened the fistula workforce, and facilitated post-operative follow-up and reintegration support for women. This integrated approach is an effective and replicable model for building capacity to deliver comprehensive fistula care services in countries where the burden of fistula is high and success rates are low (116). In such cases, women with fistulas might have access to early treatment and repeat trials for failed surgical closure. Repeat trials have the potential to achieve successful surgical closure (69, 102) and might, therefore, help improve the overall rate of satisfactory surgical closure of obstetric fistula in Africa.

## Data Availability

The raw data supporting the conclusions of this article will be made available by the authors, without undue reservation.

## Supplementary material

The supplementary material consists of separate figures showing funnel plots of publication bias tests among the studies conducted on successful surgical closure of overall obstetric fistula, VVF, RVF, VVF successfully closed and incontinent (wet), VVF successfully closed but continent (dry) obstetric VVF, and combined VVF and RVF obstetric fistula. Additionally, figures showing forest plots of the pooled rate of successful surgical closure of obstetric fistulas based on sub-regional distribution for all types of fistulas are presented in the supplementary material. Furthermore, trim and fill analysis of studies conducted on successful surgical closure of overall obstetric fistula is available in the supplementary material.
