# Supplementary material for: Successful surgical closure and continence rate of obstetric fistula in Africa: systematic review and meta-analysis
Source: Front Glob Womens Health. 2023 Oct 3;4:1188809. doi: 10.3389/fgwh.2023.1188809 (PMC10579803; doi:10.3389/fgwh.2023.1188809)
Supplement: Supplementary file 1 [file Datasheet1.docx]

**Supplementary file**

**Search strategy**

Searching was done by using Medical Subject Heading (MeSH) terms related with successful fistula closure or repair. MeSH terms enables us to select related research articles. We conducted the search for terms using Boolean operators "AND" and "OR," both separately and in combinations. The search terms for rate of successful fistula closure was ((Fistula) OR (Obstetrics Fistula)) OR (Urinary Fistula)) OR (Vesicovaginal fistula)) OR (Ureterovaginal fistula)) OR (Rectovaginal fistula)) AND (Intervention)) OR (surgical Repair)) OR (surgical Closure)) OR (outcomes)) OR (Successful surgical closure outcomes)) AND (Algeria) or (Angola) or (Benin) or (Botswana) or (Burkina Faso) or (Burundi) or (Cabo Verde) or (Cameroon) or (Central African Republic) or (Chad) or (Comoros) or (Congo, Democratic Republic of the Congo ) or (Cote d'Ivoire) or (Djibouti) or (Egypt) or (Equatorial Guinea) or (Eritrea) or (Eswatini) or (Ethiopia) or (Gabon) or (Gambia) or (Ghana) or (Guinea) or (Guinea-Bissau) or (Kenya) or (Lesotho) or or (Liberia) or (Libya) or (Madagascar) or (Malawi) or (Mali) or (Mauritania) or (Mauritius) or (Morocco) or (Mozambique) or (Namibia) or (Niger) or (Nigeria) or (Rwanda) or (Sao Tome and Principe) or (Senegal) or (Seychelles) or (Sierra Leone) or (Somalia) or (South Africa) or (South Sudan) or (Sudan) or (Tanzania) or (Togo) or (Tunisia) or (Uganda) or (Zambia) or (Zimbabwe) or (Africa).

Figure 1: Funnel plot of publication bias test among the studies conducted on successful surgical closure of overall obstetric fistula in Africa

Figure 2: Trim and fill analysis of studies conducted on successful surgical closure of overall obstetric fistula in Africa

Figure 3: Forest plot of the pooled rate of successful surgical closure of overall obstetric fistula based on regional distribution in Africa

Figure 4: Funnel plot of publication bias test among the studies conducted on successful surgical closure of obstetric VVF in Africa

Figure 5: Forest plot of the pooled rate of successful surgical closure of obstetric VVF with unknown status of incontinence based on regional distribution in Africa

Figure 6: Funnel plot of publication bias test among the studies conducted on successfully closed and continent (dry) surgical closure of obstetric VVF in Africa

Figure 7: Funnel plot of publication bias test among the studies conducted on successfully closed and incontinent (wet) surgical closure of obstetric VVF in Africa

Figure 8: Forest plot of the pooled rate of successfully closed and continent (dry) obstetric VVF based on regional distribution in Africa

Figure 9: Forest plot of the pooled rate of successfully closed and incontinent (wet) obstetric VVF based on regional distribution in Africa

Figure 10: Funnel plot of publication bias test among the studies conducted on surgical closure of obstetric RVF in Africa

Figure 11: Funnel plot of publication bias test among the studies conducted on surgical closure of combined VVF and RVF in Africa

Table 1: The Joanna Briggs Institute assessment of included studies

| No | Author | Title | Publication Year 1 | JBI Score |
| --- | --- | --- | --- | --- |
|  | Katie Tayler-Smith et al. | Obstetric Fistula in Burundi: a comprehensive approach to managing women with this neglected disease | 2013 | 7 |
|  | Anne-Caroline Benski et al. | Prognostic factors and long-term outcomes of obstetric fistula care using the Tanguiéta model | 2020 | 6 |
|  | SOLBJØRG SJØVEIAN et al. | Surgical outcome of obstetric fistula: a retrospective analysis of 595 patients | 2021 | 8 |
|  | Michel Mpunga Mafu et al. | Factors associated with surgical repair success of female genital fistula in the Democratic Republic of Congo: Experiences of the Fistula Care Plus Project, 2017–2019 | 2022 | 8 |
|  | Raha Maroyi et al. | The mobile surgical outreach program for management of patients with genital fistula in the Democratic Republic of Congo | 2020 | 7 |
|  | Justin L Paluku et al. | Obstetric vesico-vaginal fistulae seen in the Northern Democratic Republic of Congo: a descriptive study | 2016 | 8 |
|  | Tom J. I. P. Raassen et al. | Prospective results after first-time surgery for obstetric fistulas in East African women | 2007 | 7 |
|  | Bassem S. Wadie et al. | Repair of vesicovaginal fistula: Single-centre experience and analysis of outcome predictors | 2011 | 6 |
|  | Judith T. | Predicting the risk of failure of closure of obstetric fistula and residual urinary incontinence using a classification system | 2008 | 7 |
|  | Andrew Browning | Characteristics, management, and outcomes of repair of rectovaginal fistula among 1100 consecutive cases of female genital tract fistula in Ethiopia | 2015 | 6 |
|  | Shimelis Tadesse et al. | Obstetric fistula repair failure and its  associated factors among women underwent repair in Yirgalem Hamlin fistula center, Sidama Regional State, Southern Ethiopia, 2021: a retrospective cross sectional study | 2022 | 8 |
|  | A Browning et al | Risk factors for developing residual urinary  incontinence after obstetric fistula repair | 2006 | 7 |
|  | Rahel Nardos et al | Risk factors that predict failure after vaginal repair of obstetric vesicovaginal fistulae | 2008 | 7 |
|  | Demisew Anemu et al | Characteristics and repair outcome  of patients with Vesicovaginal fistula  managed in Jimma University teaching  Hospital, Ethiopia | 2016 | 7 |
|  | Alexandre Delamou et al | Fistula recurrence, pregnancy, and childbirth following successful closure of female genital fistula in Guinea: a longitudinal study | 2017 | 6 |
|  | Alexandre Delamou et al | Good clinical outcomes from a 7-year holistic programme of fistula repair in Guinea | 2015 | 6 |
|  | Laurence Bernard et al | Predictors of Obstetric Fistula Repair  Outcomes in Lubango, Angola | 2019 | 8 |
|  | Matthieu Loposso et al | Predictors of Recurrence and Successful Treatment Following Obstetric Fistula Surgery | 2016 | 8 |
|  | Matthieu Nkumu Loposso | Obstetric Fistula in a District Hospital in DR Congo: Fistula Still Occur Despite Access to Caesarean Section | 2015 | 7 |
|  | AMREEN HUSAIN et al | Surgical Management of Complex Obstetric Fistula in Eritrea | 2005 | 8 |
|  | M. Muleta et al | Obstetric fistulae: a retrospective study  of 1210 cases at the Addis Ababa Fistula  Hospital | 2015 | 7 |
|  | Abera Molla et al | Survival Modeling on the Determinants of Time to Recovery from Obstetric Fistula: The Case of Mekelle Hamlin Fistula  Center, Ethiopia | 2022 | 7 |
|  | A Browning et al | Women with obstetric fistula in Ethiopia:  a 6-month follow up after surgical treatment | 2008 | 6 |
|  | Christine Murray et al | Urinary and faecal incontinence following delayed primary repair of obstetric genital fistula | 2002 | 8 |
|  | Tesfaye Getachew et al | Survival Analysis of Time to Recovery from Obstetric Fistula: A Case Study at Yirgalem Hamlin Fistula Hospital, Ethiopia | 2015 | 8 |
|  | Alexandre Delamou et al | Factors associated with the failure of  obstetric fistula repair in Guinea:  implications for practice | 2016 | 7 |
|  | Lesley Hawkins et al | Characteristics and surgical success of patients presenting for repair of obstetric  fistula in western Kenya | 2013 | 8 |
|  | J. Kelly et al | Epidemiologic Study of Vesicovaginal Fistulas in Ethiopia | 1993 | 7 |
|  | Erin McFadden et al. | Retrospective Review of Predisposing Factors and Surgical Outcomes in Obstetric Fistula Patients at a Single Teaching Hospital in Western Kenya | 2010 | 6 |
|  | Oxana Munoz et al. | Factors Influencing Post-Operative Short-Term Outcomes of Vesicovaginal Fistula Repairs in a Community Hospital in Liberia | 2011 | 6 |
|  | Itengre Ouedraogo et al. | Obstetric fistula in Niger: 6-month postoperative follow-up of 384 patients from the Danja Fistula Center | 2017 | 8 |
|  | Marcella L et al. | Repair of obstetric vesicovaginal fistulas in Africa | 2006 | 6 |
|  | G.S. Melah et al. | Early versus late closure of vesicovaginal fistulas | 2006 | 8 |
|  | I. O. Morhason-Bello et al. | Uncomplicated midvaginal vesico-vaginal fistula repair in Ibadan: a comparison of the abdominal and vaginal routes | 2008 | 8 |
|  | Carolyn V. Kirschner et al. | Obstetric fistula: the ECWA Evangel VVF Center surgical experience from Jos, Nigeria | 2010 | 7 |
|  | K. Waaldijka et al. | Surgical classification of obstetric fistulas | 1995 | 6 |
|  | K. Waaldijk et al. | The imediate surgical management of fresh obstetric fistulas with catheter and/or early closure | 1994 | 7 |
|  | P. Hilton et al. | Epidemiological and Surgical Aspects of Urogenital Fistulae: A Review of 25 Years' Experience in Southeast Nigeria | 1998 | 6 |
|  | Tekle G. Egziabher. | Obstetric fistula management and predictors of successful closure among women attending a public tertiary hospital in Rwanda: a retrospective review of records | 2015 | 8 |
|  | Alyona Lewis et al. | Genitourinary Fistula Experience in Sierra Leone: Review of 505 Cases | 2009 | 7 |
|  | Lauren Holt, et al | Risk factors for early and late failures following repair of urogenital fistulas | 2020 | 8 |
|  | Musa Kayondo et al. | Predictors and outcome of surgical repair of obstetric fistula at a regional referral hospital, Mbarara, western Uganda | 2011 | 7 |
|  | A Holme,a et al. | Obstetric fistulae: a study of women managed at the Monze Mission Hospital, Zambia | 2007 | 8 |
|  | A. John Tomlinson | A randomised controlled trial of antibiotic prophylaxis for vesico-vaginal fistula repair | 1998 | 8 |
|  | Leon Mubikayi et al. | Determinants of Outcomes and Prognosis Score in Obstetric Vesico-Vaginal Fistula Repair | 2017 | 8 |
|  | Mathias Onsrud et al. | Cesarean delivery-related fistulae in the Democratic Republic of Congo | 2011 | 6 |
|  | Hassan Shaker et al. | Obstetric Vesico-Vaginal Fistula Repair:  Should We Trim The Fistula Edges?  A Randomized Prospective Study | 2011 | 7 |
|  | Leltework Yismaw et al | Time to recovery from obstetric fistula and determinants in Gondar university teaching and referral hospital, northwest Ethiopia | 2019 | 8 |
|  | Million Wesenu Demissie et al. | Determinants and Heterogeneity of Time -to- Recovery from Obstetric Fistula Patients; Comparison of Acceleration Failure Time and Parametric Shared Frailty Models | 2017 | 8 |
|  | Moudouni S et al. | Obstetrical vesico-vaginal fistula. Report of 114 cases | 2001 | 6 |
|  | Aniefiok J. Umoiyoho et al. | Obstetric Fistula Repair: Experience with Hospital-Based Outreach Approach in Nigeria | 2012 | 7 |
|  | C. O. Chigbu et al. | Juxtacervical vesicovaginal fistulae: Outcome by route of repair | 2006 | 6 |
|  | Temitope Omoladun Okunola et al. | Profile and outcome of patients with recurrent urogenital fistula in a fistula centre in Nigeria | 2018 | 7 |
|  | Rufus Wale Ojewola et al. | Transabdominal Repair of Vesicovaginal Fistulae: A 10‑Year Tertiary Care Hospital Experience in Nigeria | 2018 | 7 |
|  | Eric Bohoussou et al | Results of the Management of Urogenital  Fistulae from Community Caravans | 2017 | 7 |
|  | J TUKUR et al | The contribution of Gishiri cut to vesicovaginal fistula in Birnin Kudu, Northern Nigeria | 2006 | 6 |
|  | Yasam Kemal Akpak et al | Evaluation of etiology, characteristics, and treatment of patients with vesicovaginal fistula observed in rural Africa | 2019 | 7 |
|  | Fiona Katherine McCurdie et al | Vesicovaginal fistula in Uganda | 2018 | 7 |
|  | Yves-Jacques Castille et al. | Impact of a program of physiotherapy and health education on the outcome of obstetric fistula surgery | 2014 | 8 |
|  | S. WAD~L-ZWAN et al. | A REVIEW OF URINARY FISTULAE IN A UNIVERSITY TEACHING HOSPITAL | 1983 | 7 |
|  | MOHSEN AYED et al. | Prognostic factors of recurrence after vesicovaginal fistula repair | 2006 | 6 |
|  | Judith T et al. | Predicting the risk of failure of closure of obstetric fistula and residual urinary incontinence  using a classification system | 2008 | 7 |
|  | Sultan Hussen et al. | Time-to-recovery from obstetric fistula and associated factors: The case of Harar Hamlin Fistula Center | 2017 | 7 |
|  | Rahel Nardos et al. | Duration of bladder catheterization after surgery for obstetric fistula | 2008 | 8 |
|  | Samuel Hailu | Predictors of Time to Recovery from a Surgical Repair of Obstetric Fistula at Mekelle Hamlin Fistula Center, Tigray, Ethiopia | 2017 | 8 |
|  | G. *C .* BIR et al. | OBSTETRIC VESICO-VAGINAL AND ALLIED FISTULAE A Report on 70 Cases | 1967 | 5 |
|  | I. Nafiou et al | Obstetric vesico-vaginal fistulas at the National Hospital of Niamey, Niger | 2007 | 6 |
|  | Kees Waaldijk et al. | The immediate management of fresh obstetric fistulas | 2004 | 6 |
|  | Thomas E et al. | Surgery for the obstetric vesicovaginal fistula: A review of 100 operations in 82 patients | 1993 | 5 |
|  | Pierre Marie Tebeu et al. | Surgical outcome of genito-urinary obstetric fistulas (GUOF) with or without bladder neck involvement: an experience from the University Teaching Hospital,  Yaoundé, Cameroon | 2019 | 6 |
|  | Mark A. Barone et al. | Determinants of Postoperative Outcomes of Female Genital Fistula Repair Surgery | 2012 | 7 |
|  | Judith T.W et al. | Genital Tract Fistula Repair on 116 Women | 1998 | 6 |
|  | Y. Rijken et al. | Urogenital and recto-vaginal fistulas in southern Malawi: A report on 407 patients | 2007 | 6 |
|  | Amanuel Gessessew et al. | Genitourinary and rectovaginal fistulae in Adigrat Zonal Hospital, Tigray, north Ethiopia | 2003 | 7 |
|  | Munir-deen et al. | Epidemiology of Vesico-Vaginal Fistula at the University of Ilorin Teaching hospital,Ilorin, Nigeria | 2002 | 8 |
|  | STEVEN D. et al. | GENITOURINARY RECONSTRUCTION IN OBSTETRIC FISTULAS | 1994 | 5 |
|  | John Lawson | Tropical obstetrics and gynaecology. 3. Vesico-vaginal fistula-a tropical  disease | 1989 | 6 |
|  | Katherine T. et al. | SHORT-TERM COMPLICATIONS AND ASSOCIATED FACTORS AMONG WOMEN UNDERGOING OBSTETRIC FISTULA REPAIR AT BUGANDO MEDICAL CENTRE, MWANZA, TANZANIA | 2016 | 6 |
|  | Abdoulaye Bobo Diallo et al. | Vesicovaginal Fistulas: Anatomical Clinical and Surgical Aspects in the Conakry University Hospital Center | 2015 | 8 |
|  | Brian Hancock MD FRCS et al. | Vesicovaginal Fistula Surgery in Uganda | 2004 | 7 |
|  | Raha Maroyi et al | Obstetric vesico-vaginal fistulas: Mid vaginal and juxtacervical fistula repair  outcomes in the Democratic Republic of Congo (Clinical article) | 2020 | 7 |
|  | Aynie, Amare A. et al | Magnitude of repair failure and associated factors among women undergone obstetric fistula repair in Bahir Dar Hamlin Fistula Center, Amhara Region, Northwest Ethiopia | 2019 | 8 |
|  | MULU MULETA et al | Obstetric fistula in 14,928 Ethiopian women | 2010 | 8 |
|  | Tesfay Yohannes Ambese et al | Effect of vaginal scarring on the recovery of surgical repair of obstetric fistula in Northern Ethiopia | 2022 | 7 |
|  | Steven N. Shephard et al | Effect of HIV infection on outcomes after surgical repair of genital fistula | 2017 | 7 |

| Table 2: Data extraction sheet for the study | | | | | | | | | | | |
| --- | --- | --- | --- | --- | --- | --- | --- | --- | --- | --- | --- |
| Authors | Publication Year | Study design | Country | Sub-region | Sample size | Successful closure of obstetrics fistula | VVF Closure rate with unknown status | Rate of VVF Closed & dry | Rate of VVF Closed & incontinent | RVF  Closure rate | Combined VVF&RVF closure rate |
| Laurence Bernard et al. | 2019 | RCS | Angola | Central Africa | 243 | 42 |  |  |  |  |  |
| A. John Tomlinson | 1998 | RCS | Benin | West Africa | 79 |  |  | 66 |  |  |  |
| Anne-Caroline Benski et al. | 2019 | RCS | Benin | West Africa | 308 |  | 85.3 | 66.6 | 19 |  |  |
| Katie Tayler-Smith et al. | 2013 | RCS | Burundi | East Africa | 454 | 87 |  |  |  |  |  |
|  |  |  |  |  | 395 |  | 86.3 | 65.3 | 21 |  |  |
|  |  |  |  |  | 41 |  |  |  |  | 92.7 |  |
| Pierre Marie Tebeu et al. | 2019 | RCS | Cameroon | West Africa | 92 |  |  | 68.47 |  |  |  |
| Eric Bohoussou et al. | 2017 | RCT | Cote d’Ivoire | West Africa | 266 |  |  | 70 |  |  |  |
| Justin L Paluku et al. | 2015 | RCS | DR Congo | Central Africa | 163 |  | 90.2 | 87.1 | 2.7 |  |  |
| Leon Mubikayi et al. | 2017 | PCS | DR Congo | Central Africa | 483 | 85.7 |  |  |  |  |  |
|  |  |  |  |  | 470 |  | 85.6 | 83 | 2.6 |  |  |
| Mathias Onsrud et al. | 2011 | RCS | DR Congo | Central Africa | 576 | 83.07 |  |  |  |  |  |
|  |  |  | DR Congo | Central Africa | 400 |  | 82.5 | 69.75 | 12.75 |  |  |
| Matthieu Loposso et al. | 2016 | PCS | DR Congo | Central Africa | 166 |  |  | 71.7 |  |  |  |
| Matthieu Nkumu Loposso | 2014 | RCS | DR Congo | Central Africa | 146 |  | 65 | 63 |  |  |  |
| Michel Mpunga Mafu et al. | 2022 | RCS | DR Congo | Central Africa | 895 | 92 | 92 | 88 | 12 |  |  |
| Raha Maroyi et al. | 2021 | RCS  RCS | DR Congo | Central Africa | 450 |  |  | 96 | 2.8 |  |  |
| Raha Maroyi et al. | 2020 |  | DR Congo | Central Africa | 1413 | 92.6 | 97.9 | 92.6 | 5.3 |  |  |
| SOLBJØRG SJØVEIAN et al. | 2011 | RCS | DR Congo | Central Africa | 595 | 87.1 |  |  | 15.6 |  |  |
| Tom J. I. P. Raassen et al. | 2021 | PCS | E/Africa | East Africa | 565 |  | 90.7 | 82.3 | 8.4 |  |  |
| Judith T W goh, et al | 2021 | RCS | E/Africa | East Africa | 101 |  |  |  |  | 87 |  |
| Barone et al. | 2012 | RCS | E/Africa | East Africa | 1274 |  | 81.7 | 64.6 | 15.1 |  |  |
| Bassem S. Wadie et al. | 2011 | RCS | Egypt | North Africa | 100 |  | 80 |  | 5 |  |  |
| Hassan Shaker et al. | 2011 | RCT | Egypt | North Africa | 63 |  | 71.4 |  |  |  |  |
| AMREEN HUSAIN et al. | 2005 | RCS | Eritrea | East Africa | 50 | 64.5 |  |  |  |  |  |
|  |  |  |  |  | 42 |  | 63 |  |  |  |  |
| A Browning et al | 2008 | PCS | Ethiopia | East Africa | 240 |  | 97.9 |  | 24.3 |  |  |
| A Browning et al. | 2006 | RCS | Ethiopia | East Africa | 481 |  | 100 | 68 | 32 |  |  |
| Abera M et al. | 2022 | RCS | Ethiopia | East Africa | 328 | 83.39 |  |  |  |  |  |
|  |  |  |  |  | 268 |  | 86.69 |  |  |  |  |
|  |  |  |  |  | 60 |  |  |  |  |  |  |
| Amanuel Gessessew et al. | 2003 | RCS | Ethiopia | East Africa | 182 |  | 91.7 | 86.8 | 8.2 |  |  |
| Amare A. Aynie et al. | 2019 | RCS | Ethiopia | East Africa | 385 | 86 | 64.5 |  |  |  |  |
| Andrew Browning | 2015 | RCS | Ethiopia | East Africa | 933 |  | 96.2 | 78 | 19.5 |  |  |
|  |  |  |  |  | 49 |  |  |  |  | 98 |  |
|  |  |  |  |  | 79 |  |  |  |  |  | 52.8 |
| Christine Murray et al. | 1999 | RCS | Ethiopia | East Africa | 44 |  | 100 | 45 | 55 |  |  |
| J. Kelly et al. | 1993 | RCS | Ethiopia | East Africa | 309 |  | 94.2 | 88 | 6.2 |  |  |
| Judith T. W. et al | 2008 | RCS | Ethiopia | East Africa | 987 |  | 97.3 | 76.1 | 29.1 |  |  |
| Judith T.W. Goh | 19988 | RCS | Ethiopia | East Africa | 108 |  | 97.2 | 92.6 | 7.4 |  |  |
| Leltework Yismaw et al | 2019 | RCS | Ethiopia | East Africa | 612 | 88.07 |  |  |  |  |  |
| M. Muleta et al. | 2015 | RCS | Ethiopia | East Africa | 1210 | 92.6 |  |  |  |  |  |
|  |  |  |  |  | 960 |  | 97.66 |  |  |  |  |
|  |  |  |  |  | 90 |  |  |  |  | 86.11 |  |
|  |  |  |  |  | 31 |  |  |  |  |  | 60.4 |
| Million Wesenu Demissie et al. | 2017 | RCS | Ethiopia | East Africa | 270 | 81.48 |  |  |  |  |  |
| MULU MULETA et al. | 2010 | RCS | Ethiopia | East Africa | 14239 | 92.5 |  |  | 18.8 |  |  |
| R. Nardos et al | 2008 | PCS | Ethiopia | East Africa | 212 |  | 99 | 64.15 | 35.84 |  |  |
| Rahel Nardos et al. | 2009 | RCS | Ethiopia | East Africa | 189 |  | 95.2 | 82 | 16.12 |  |  |
|  |  |  |  |  | 1045 |  | 89 | 83 | 17 |  |  |
| Samuel Hailu | 2018 | RCS | Ethiopia | East Africa | 306 |  |  | 53.92 |  |  |  |
| Shimelis Tadesse et al. | 2022 | RCS | Ethiopia | East Africa | 562 |  | 92.9 |  |  |  |  |
| Sultan Hussen et al. | 2017 | RCS | Ethiopia | East Africa | 433 |  |  | 67.21 |  |  |  |
| Tesfay Yohannes Ambese et al | 2022 | RCS | Ethiopia | East Africa | 224 |  |  |  |  |  |  |
| Demisew Anemu Sori et al. | 2016 | Cross sectional | Ethiopia | East Africa | 168 |  | 93.45 | 84.5 | 10.5 |  |  |
| Tesfaye Getachew et al. | 2015 | RCS | Ethiopia | East Africa | 360 |  | 81.7 |  |  |  |  |
| Abdoulaye Bobo Diallo et al. | 2015 | RCS | Guinea | West Africa | 152 |  | 69 | 62 | 7 |  |  |
| Alexandre Delamou et al. | 2015 | RCS | Guinea | West Africa | 2116 | 84.8 |  | 79.4 | 5.6 |  |  |
|  |  |  |  |  | 48 |  |  |  |  |  | 56.3 |
| Alexandre Delamou et al. | 2016 | RCS | Guinea | West Africa | 754 | 85.5 |  |  |  |  |  |
|  |  |  |  |  | 646 |  | 86.5 | 83 | 3.5 |  |  |
|  |  |  |  |  | 92 |  |  |  |  |  | 77.2 |
| Alexandre et al. | 2017 | PRC | Guinea | West Africa | 447 |  |  | 93 | 7 |  |  |
| Erin McFadden et al. | 2011 | RCS | kenya | East Africa | 89 |  | 95 |  | 45 |  |  |
| G. C. BIRD et al. | 1967 | RCS | Kenya | East Africa | 70 |  | 83 | 70 | 13 |  |  |
| Lesley Hawkins et al.  Lesley Hawkins et al. | 2017 | RCS | Kenya | East Africa | 245 |  | 83.26 |  |  |  |  |
|  |  |  |  |  | 260 | 81.9 |  |  |  |  |  |
| Munoz et al. | 2011 | RCS and PCS | Liberia | West Africa | 40 |  | 100 | 70 | 100 |  |  |
| Y. Rijken et al. | 2007 | RCS | Malawi | East Africa | 407 | 87.9 |  |  |  |  |  |
|  |  |  |  |  | 396 |  | 98.5 | 82.3 | 16.2 |  |  |
| Moudoni S et al. | 2001 | RCS | Morocco | North Africa | 114 |  | 86.8 | 76.3 | 10.5 |  |  |
| I. Nafiou et al. | 2007 | PCS | Niger | West Africa | 104 |  | 86.6 | 73.1 | 13.5 |  |  |
| Itengre Ouedraogo et al | 2016 | PCS | Niger | West Africa | 384 |  | 70.3 | 53.9 | 16.4 |  |  |
| Marcella L et al. | 2006 | RCS | Niger | West Africa | 73 |  | 68.4 | 56 | 12.32 |  |  |
| Kees Waaldijk et al. | 2004 | RCS | Nigeria | West Africa | 1451 |  | 94.3 | 93.2 | 6.8 |  |  |
|  |  |  |  |  | 1716 | 98.5 |  |  |  |  |  |
| Aniefiok J. Umoiyoho et al. | 2012 | PCS | Nigeria | West Africa | 52 |  | 100 | 100 |  |  |  |
| C. O. CHIGBU et al. | 2009 | RCS | Nigeria | West Africa | 78 |  |  | 82.1 |  |  |  |
| Carolyn V. Kirschner et al. | 2010 | RCS | Nigeria | West Africa | 926 |  |  | 73.75 | 13.4 |  |  |
| G.S. Melah et al. | 2006 | PCS | Nigeria | West Africa | 80 |  | 100 | 96.25 | 3.7 |  |  |
| I. O. Morhason-Bello et al. | 2008 | RCS | Nigeria | West Africa | 71 |  |  | 79.2 |  |  |  |
| J TUKUR et al. | 2006 | RCS | Nigeria | West Africa | 34 |  | 76.5 |  |  |  |  |
| John Lawson | 1989 | RCS | Nigeria | West Africa | 377 |  | 71.3 |  |  |  |  |
| K. Waaldijk | 1994 | RCS | Nigeria | West Africa | 170 |  | 91.8 | 85.88 | 5.88 |  |  |
| K. Waaldijka et al. | 1995 | RCS | Nigeria | West Africa | 650 |  | 96.92 | 87.23 | 9.69 |  |  |
| P. Hilton et al. | 1998 | RCS | Nigeria | West Africa | 1954 | 97.7 |  |  |  |  |  |
| Rufus Wale Ojewola et al. | 2018 | RCS | Nigeria | West Africa | 53 |  | 88.7 |  |  |  |  |
| Steven N. Shephard et al. | 2017 | RCS | Nigeria | West Africa | 2677 |  | 68.2 | 55.78 |  |  |  |
|  |  |  |  |  | 283 |  |  |  |  | 85.9 |  |
| Munir deen et al. | 2002 | RCS | Nigeria | West Africa | 44 |  | 87.9 |  |  |  |  |
| Temitope Omoladun Okunola et al. | 2018 | RCS | Nigeria | West Africa | 154 |  | 77.3 | 64.3 | 13 |  |  |
| Tekle G. Egziabher. | 2015 | RCS | Rwanda | East Africa | 272 | 86.3 |  |  |  |  |  |
|  |  |  |  |  | 93 |  |  | 84.9 |  |  |  |
|  |  |  |  |  | 48 |  |  |  |  | 95.8 |  |
| Alyona Lewis et al. | 2009 | RCS | Sierra Leone | West Africa | 435 |  | 84.3 |  |  |  |  |
| Yasam Kemal Akpak et al. | 2020 | RCS | Sudan | East Africa | 51 |  | 84.3 |  |  |  |  |
| [Katherine T. Magali et al.](https://www.emjema.org/index.php/EMJ/search/search?field=author&criteria=Katherine%20T.%20Magali) | 2017 | PCS | Tanzania | East Africa | 132 | 86.3 |  |  |  |  |  |
| MOHSEN AYED et al | 2006 | RCS | Tunisia | North Africa | 73 |  | 86.7 |  |  |  |  |
| Fiona Katherine McCurdie, et al | 2018 | RCS | Uganda | East Africa | 93 |  |  | 94 |  |  |  |
| Brian Hancock et al. | 2004 | PCS | Uganda | East Africa | 298 | 93.53 |  |  | 3.2 |  |  |
|  |  |  |  |  | 74 |  | 94 |  |  |  |  |
| Lauren Holt et al. | 2021 | RCS | Uganda | East Africa | 541 |  | 89.1 |  |  |  |  |
| Musa Kayondo et al. | 2011 | PCS | Uganda | East Africa | 77 | 77.9 |  |  |  |  |  |
|  |  |  |  |  | 69 |  | 79.7 | 76.4 | 23.6 |  |  |
| Yves-Jacques Castille et al. | 2013 | RCS | W/Africa | West Africa | 211 |  | 66.7 |  |  |  |  |
| Thomas E. Elkins et al. | 1993 | RCS | W/Africa | West Africa | 80 |  | 95 |  |  |  |  |
| A Holme,a et al. | 2007 | RCS | Zambia | East Africa | 252 |  | 90.1 | 72.6 | 9.9 |  |  |
| S. WAD~L-ZWAN et al. | 1983 | RCS | Zambia | East Africa | 44 |  |  | 59.1 |  |  |  |

NB: RCS: Petrospective cohort study, PCS: Prospective cohort study and RCT: Randomized control trail
